# A Unique Heterozygous *CARD11* Mutation Combines Pathogenic Features of Both Gain- and Loss-of-Function Patients in a Four-Generation Family

**DOI:** 10.3389/fimmu.2018.02944

**Published:** 2018-12-12

**Authors:** Marylin Desjardins, Swadhinya Arjunaraja, Jeffrey R. Stinson, Batsukh Dorjbal, Janani Sundaresan, Julie Niemela, Mark Raffeld, Helen F. Matthews, Angela Wang, Pamela Angelus, Helen C. Su, Bruce D. Mazer, Andrew L. Snow

**Affiliations:** ^1^Division of Allergy and Immunology, Department of Paediatrics, McGill University Health Centre, Montreal, QC, Canada; ^2^Meakins-Christie Laboratories of the Research Institute of the McGill University Health Centre, Montreal, QC, Canada; ^3^Department of Pharmacology and Molecular Therapeutics, Uniformed Services University of Health Sciences, Bethesda, MD, United States; ^4^Department of Laboratory Medicine, NIH Clinical Center, Bethesda, MD, United States; ^5^Laboratory of Pathology, Center for Cancer Research, National Cancer Institute, National Institutes of Health, Bethesda, MD, United States; ^6^Laboratory of Immune System Biology, National Institute of Allergy and Infectious Diseases, National Institutes of Health, Bethesda, MD, United States; ^7^Laboratory of Clinical Immunology and Microbiology, National Institute of Allergy and Infectious Diseases, National Institutes of Health, Bethesda, MD, United States; ^8^Clinical Research Directorate/Clinical Monitoring Research Program, Leidos Biomedical Research, Inc., National Cancer Institute at Frederick, Frederick, MD, United States

**Keywords:** CARD11, BENTA, Atopy, B cell lymphocytosis, primary immumunodeficiencies

## Abstract

CARD11 is a lymphocyte-specific scaffold molecule required for proper activation of B- and T-cells in response to antigen. Germline gain-of-function (GOF) mutations in the *CARD11* gene cause a unique B cell lymphoproliferative disorder known as B cell Expansion with NF-κB and T cell Anergy (BENTA). In contrast, patients carrying loss-of-function (LOF), dominant negative (DN) *CARD11* mutations present with severe atopic disease. Interestingly, both GOF and DN CARD11 variants cause primary immunodeficiency, with recurrent bacterial and viral infections, likely resulting from impaired adaptive immune responses. This report describes a unique four-generation family harboring a novel heterozygous germline indel mutation in *CARD11* (c.701-713delinsT), leading to one altered amino acid and a deletion of 4 others (p.His234_Lys238delinsLeu). Strikingly, affected members exhibit both moderate B cell lymphocytosis *and* atopic dermatitis/allergies. Ectopic expression of this *CARD11* variant stimulated constitutive NF-κB activity in T cell lines, similar to other BENTA patient mutations. However, unlike other GOF mutants, this variant significantly impeded the ability of wild-type CARD11 to induce NF-κB activation following antigen receptor ligation. Patient lymphocytes display marked intrinsic defects in B cell differentiation and reduced T cell responsiveness *in vitro*. Collectively, these data imply that a single heterozygous *CARD11* mutation can convey both GOF and DN signaling effects, manifesting in a blended BENTA phenotype with atopic features. Our findings further emphasize the importance of balanced CARD11 signaling for normal immune responses.

## Introduction

BENTA disease [B cell Expansion with nuclear factor kappa B (NF-κB) and T cell Anergy] is a rare immunodeficiency disorder that presents with splenomegaly and unusual peripheral blood lymphocytosis comprised of naïve and immature B cells (1, 2). BENTA is caused by germline, heterozygous GOF mutations in *CARD11*, which encodes a large multi-domain scaffold molecule best known for connecting antigen receptor (AgR) ligation to NF-κB activation in B and T lymphocytes. Similar to somatic GOF mutations typically found in the coiled-coil (CC) domain of CARD11 and associated with diffuse large B-cell lymphoma (3), BENTA CARD11 mutants spontaneously aggregate to form active signaling clusters with BCL-10 and MALT1 (CBM complex), resulting in constitutive NF-κB activation without requiring B or T cell receptor interaction (4). NF-κB drives the expression of pro-survival genes in transitional and naïve B-cells to confer enhanced survival, while paradoxically rendering T cells less responsive to TCR stimulation with reduced IL-2 production (5–7). Despite the striking expansion of polyclonal B cells, BENTA patients exhibit reduced B cell memory and poor antibody responses to specific vaccines, due in part to intrinsic B cell defects in plasma cell differentiation (8). Moreover, mildly anergic T cell responses may explain increased susceptibility to certain viral infections [e.g., molluscum contagiousum, Epstein-Barr virus (EBV)]. Together, these immune abnormalities increase patients' susceptibility to viral and bacterial infections, including chronic EBV infection, and may to contribute to a higher potential risk of B-cell malignancy (9).

More recently, heterozygous, hypomorphic CARD11 mutations were uncovered in patients with elevated circulating IgE, severe atopic dermatitis, and other allergic manifestations (e.g., rhinitis, asthma, food allergies) (10–12). Strikingly, these variants dominantly interfere with wild-type (WT) CARD11 signaling to both NF-κB and mTORC1, contributing to defective T cell responses that skew toward a T helper 2 (Th2) profile consistent with atopy. Most *CARD11* DN patients also share signs of immunodeficiency that overlap with those noted in BENTA patients, including frequent sinopulmonary bacterial infections and viral infections (e.g., molluscum), and fewer class-switched memory B cells. However, B cell lymphocytosis was not observed in this cohort. Furthermore, atopic disease has not been described to date in BENTA patients.

## Case Presentation and Results

The index patient (IV.1) was referred to our dedicated immunology clinic (McGill University Health Centre-MUHC) at 18 months of age for evaluation of dermatitis and recurrent infections. He was treated with oral antibiotics at 5, 8, and 13 months of age for balanitis. He also suffered from chest X-ray-documented pneumonia and an acute otitis media, which occurred at 6 and 12 months of age, respectively. At 14 months, he was admitted to the hospital for febrile neutropenia with pneumonia and treated with intravenous antibiotics. The neutropenia resolved, but marked persistent lymphocytosis persisted. Small reactive lymphocytes were present on the peripheral blood smear. The physical examination revealed dry lichenified erythematous skin lesions with no palpable lymphadenopathy and no appreciable splenomegaly. Dermatology confirmed a diagnosis of pustular psoriasis, which was complicated by a superimposed recurrent *molluscum contagiosum* affecting his abdomen, back, perianal and leg areas as well as streptococcal perianal dermatitis.

Initial blood analyses performed at MUHC in 2012 showed a lymphocyte count of 20.4 × 10^9^/L and composed predominantly of CD19^+^ B-cells (12.4 × 10^9^/L, 61.0%), with a decreased proportion of CD3^+^ T-cells (6.7 × 10^9^/L, 33.0%) and normal CD16^+^CD56^+^ NK-cells (0.6 × 10^9^/L, 3.0%). An extended flow cytometry panel performed in 2015 (MUHC) revealed increased proportions of naïve CD19^+^sIgM^+^ B-cells (24.9 × 10^8^/L, 72.0%) and CD19^+^CD5^+^ B-cells (20.0 × 10^8^/L, 58.0%). The proportion of class-switched CD19^+^CD27^+^IgD^−^memory B-cells (0.2 × 10^8^/L, 0.6%) was decreased. IgH gene rearrangement analysis demonstrated a polyclonal B-cell lymphocytosis pattern (Supplementary Figure 1). The patient had mild hypogammaglobulinemia (IgG 3.1 g/L) associated with slightly decreased serum IgA levels (0.19 g/L), absent serum IgM (< 0.25 g/L) and normal IgE levels (63 μg/L). IgE levels increased to 444 μg/L (normal 0–240 μg/L) later when the patient reached 4 years of age. Although, the patient received all recommended vaccines for age (including live attenuated vaccines against measles, mumps, rubella and varicella), he had no specific antibody responses against *Haemophilus influenzae type B* (IgG < 0.1 μg/mL) and *Streptococcus pneumoniae* (0/14 IgG serotypes were above 1.3 μg/mL). The specific antibody responses were normal for tetanus (IgG above 4.0 IU/mL) and diphtheria toxoids (IgG above 3.0 IU/mL). Lymphocyte proliferation responses to phytohemagglutinin, concanavalin A, and anti-CD3 were also normal. Pokeweed mitogen proliferative response was decreased (stimulation index of 23.2 compared to 85.6 in healthy control). Mild splenomegaly (8.5 cm) was confirmed on abdominal ultrasound. The CT scan showed small bilateral cervical lymph nodes. Blood serology and PCR for EBV and cytomegalovirus (CMV) were negative. The patient was treated with weekly subcutaneous immunoglobulin (scIg) injection therapy (equivalent to 0.65 g/kg/month). At 5 years of age, he suffered from two consecutive pneumonias with the second episode associated with rhinovirus infection, which required hospitalization and oxygen supplementation. Among other relevant medical problems, he is known for mild bilateral conductive hearing loss and atopy IgE-mediated milk allergy (milk specific IgE: 9.95 IU/L and positive skin prick test to cow's milk extract at 2 years old) and allergic rhinitis to birch and cat.

**Figure 1:**
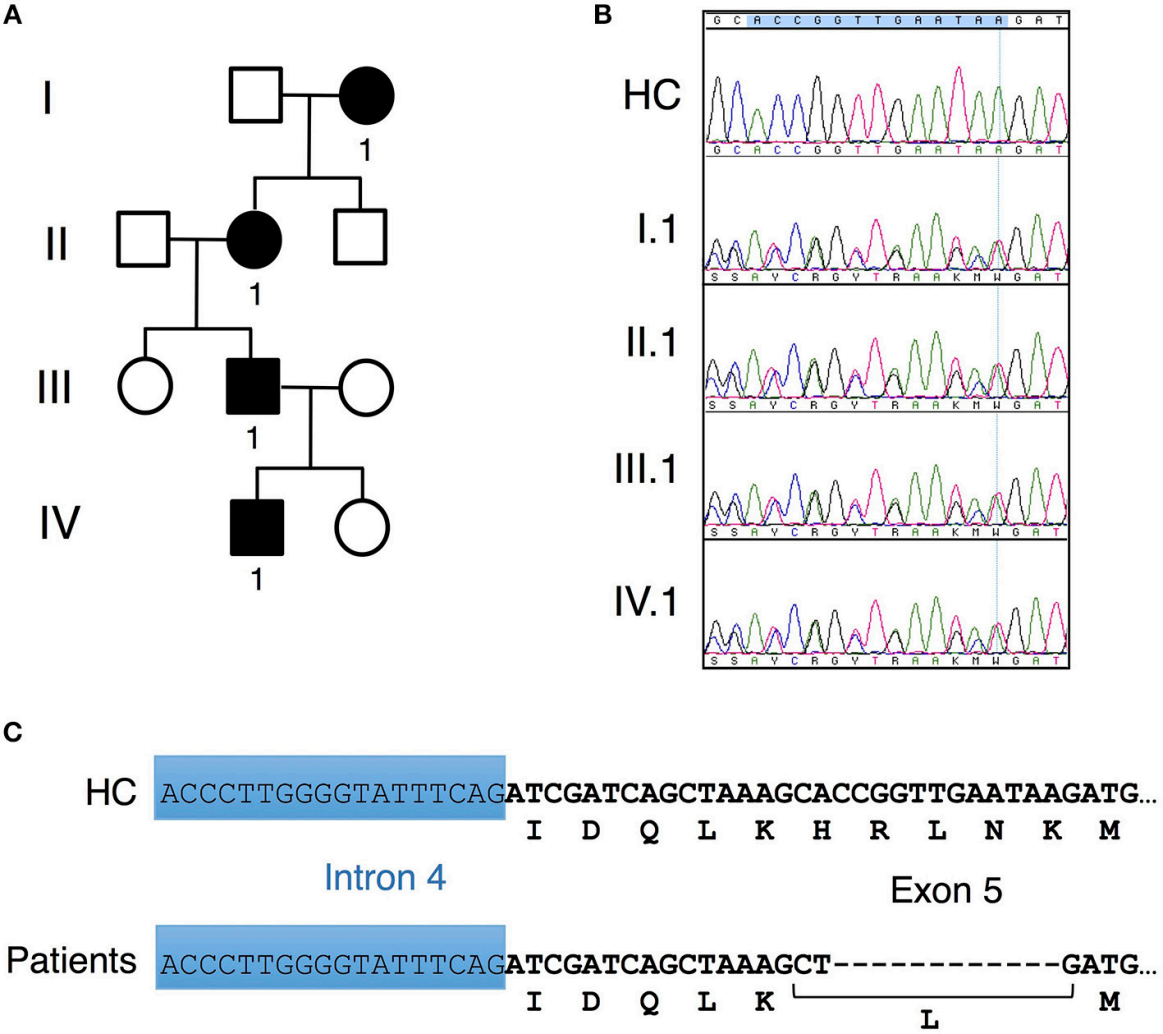
Unique *CARD11* mutation in a four-generation family. **(A)** Pedigree of patients in a four-generation BENTA family. Squares and circles represent males and females, respectively, and black represents affected patients. **(B)** Sanger DNA sequencing showing a novel in-frame 12 bp deletion in genomic DNA derived from PBMC isolated from patients II.1, III.1 and IV.1 compared to healthy control (HC). **(C)** Schematic diagram showing the nucleotide and corresponding amino acid changes comprising the H234LΔ235-8 CARD11 variant found in patients vs. healthy control.

The family reported that parents were not related and were of Caucasian origin (Figure 1A). The father (Patient III.1, 29 years old) was investigated as a child for splenomegaly, atopic dermatitis, mild intermittent asthma, molluscum contagiosum (on buttocks, legs, and arms from age 1–5 years old) and “high total B-cell number.” No diagnosis was established at the time. Over the years, he developed recurrent warts and persistent finger onychomycosis (since age 4–5). He had chickenpox at 11 years old followed by shingles as a young adult. More recently, he was treated with intravenous antibiotic for recurrent knee effusions and recurrent folliculitis of the forearms and thighs. *Staphylococcus aureus* was isolated on the initial bacterial culture. Further immunological investigations revealed a polyclonal B-cell lymphocytosis (absolute lymphocyte count of 3.4 × 10^9^/L with 33.0% of circulating CD19^+^ B-cells). B-cell subpopulation analysis also demonstrated an increased proportion of naïve CD19^+^sIgM^+^ B-cells (7.9 × 10^8^/L, 74.0%) and CD19^+^CD5^+^ B-cells (5.3 × 10^8^/L, 50.0%), while the proportion of class-switched CD19^+^CD27^+^IgD^−^memory B-cells (0.1 × 10^8^/L, 1.0%) was severely decreased. The father had a mild decrease in serum IgM level (0.48 g/L), but otherwise normal serum immunoglobulins (IgG 12.7 g/L, IgA 1.8 g/L, IgE 37–107 μg/L) and specific antibody responses against tetanus (0.9 IU/mL), diphtheria (0.6 IU/mL), and *Haemophilus influenzae type B* (HIB, 1.9 μg/mL) post vaccination. The specific humoral response against polysaccharide antigens was suboptimal with < 50% of pneumococcal serotypes above 1.3 μg/mL despite Pneumovax booster. Numbers and proportions of peripheral blood T and NK cells populations were within normal range for age. Lymphocyte mitogen proliferation assays were also normal. The father was closely monitored for the occurrence of possible lymphoproliferative disorder; he had persistently positive EBV PCR (46 774 copies/mL, EBV VCA positive ≥1/4000, EBV EBNA < 1/10) with enlarged axillary lymph nodes (up to 1.7 × 1.2 cm on the left size) and prominent fluorodeoxyglucose activity on PET-CT scan. His spleen size was at the upper limit of normal (13.3 cm). He was treated with frequent boosters of conjugated pneumococcal vaccines (e.g., Prevnar-13) and use of immunoglobulin replacement therapy was considered by the medical team.

The grandmother (II.1) reported frequent otitis externa and colds as a child. As an adult she had two pneumonias and two episodes of shingles, and was treated for Hodgkin's lymphoma (2A, mixed cellularity) at 33 years old. She has allergic rhinitis to dust and molds, frequent rhinosinusitis, persistent warts, and persistent onychomycosis. The great-grandmother (Patient I.1, 78 years old) suffered from recurrent warts, shingles and sinusitis over the years. A clinical evaluation was offered to the great grandmother at MUHC but never performed. The index patient, father and grandmother were subsequently evaluated at the NIH Clinical Center in 2015 and again in 2017. On physical exam, warts were noted on the index patient (one on toe) and father (multiple on hands). The most recent flow cytometric evaluation of PBMC confirmed expansion of naïve and immature B cells in the index patient, with few class-switched memory B cells detected (Table 1). The same pattern was evident in the father and grandmother, although circulating B cell counts decreased inversely with age. PCR indicated moderate EBV viremia in both father and grandmother (3.13 and 4.45 Log10 IU/ml). Serum IgE levels were slightly elevated in the index patient; otherwise total immunoglobulin levels were normal for all subjects. For the father, random antibody titers against rubella, measles, and VZV were normal, but absent against mumps. The grandmother showed normal antibody titers to measles, rubella, mump, tetanus and HIB, with poor responses to Pneumovax (5/23 serotypes detected).

Table 1Flow cytometric phenotyping of patient PBMC and serum Ig levels.

**Table:** 

	**II.1**		**III.1**		**IV.1**		**Normal range (adult)**	
	**%**	**#**	**%**	**#**	**%**	**#**	**%**	**#**
Total B cells	20	612	32.5	1,066	43.3	1,602	3–19	59–329
Naïve CD19+IgD+	17.7	542	30.3	994	41.3	1,528	1.4–14.4	25–324
CD19+ CD10+	4	122	14.5	476	33.3	1,232	0.1–3.4	2–76
CD19+ CD27+	0.2	6	0.1	3	0	0	0.4–2.3	5–46
CD3+ T	71.3	2,182	61.2	2,007	53	1,961	60–83.7	714–2266
CD4+ T	35.1	1,074	405	1,328	34.9	1,291	31.9–62.2	359–1565
CD8+ T	31.7	970	16.3	535	13.7	507	11.2–34.8	178–853
NK cells	8.9	272	6.5	213	4.1	152	6.2–34.6	126–729
NKT cells	10	306	6	197	3.6	133	2.2–12.4	29–299

**Table:** 

	**II.1**	**III.1**	**IV.1**
IgG	1135 (700–1600 mg/dL)	1129 (700–1600 mg/dL)	1204 (504–1465 mg/dL)
IgA	255 (70–400 mg/dL)	161 (70–400 mg/dL)	72(27–195 mg/dL)
IgM	37 (40–230 mg/dL)	39 (40–230 mg/dL)	14 (24–210 mg/dL)
IgE	16 (0–90 IU/mL)	37.5 (0–90 IU/mL)	136 (0–90 IU/mL)

*Values in blue and red color indicate lower and higher levels, respectively, compared to normal healthy control ranges listed at right. Absolute counts indicated for each cell type are per μl. Normal Ig levels corresponding to patient age groups are indicated in parentheses*.

**Figure 2:**
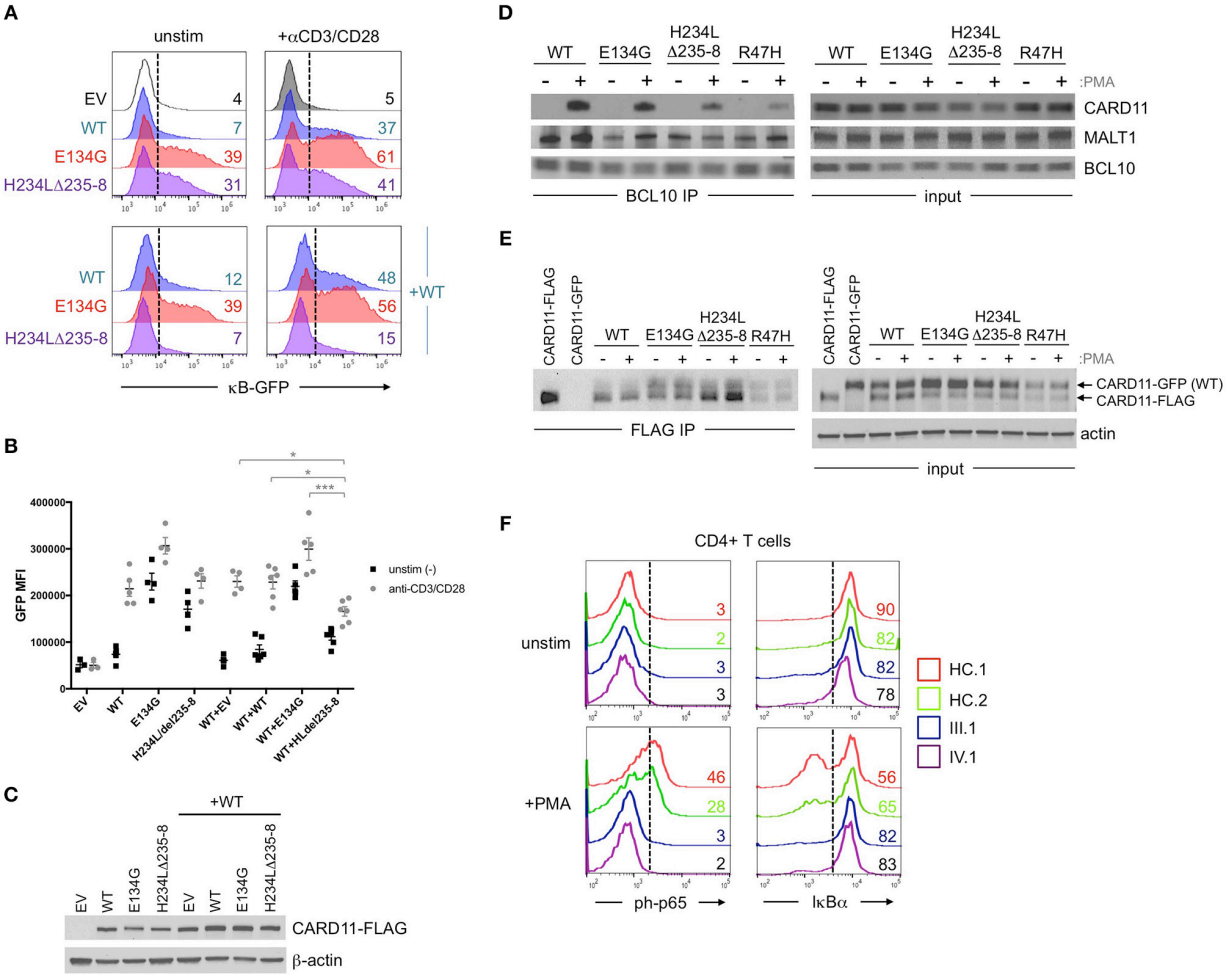
Abnormal NF-κB activation induced by H234LΔ235-8 CARD11 *in vitro*. **(A)** JPM50.6 T cells were transfected with 5 μg of either empty vector (EV), WT, E134G (GOF mutant), or H234LΔ235-8 mutant CARD11-FLAG plasmids alone (top panel) or in the presence of 5 μg WT CARD11 plasmid (bottom panel) as previously described (14). After 24 h incubation in complete RPMI, transfected cells were stimulated with 1 μg/ml of anti-CD3 and anti-CD28 Abs or left unstimulated for an additional 24 h. GFP expression (reflecting relative NF-κB activity) was subsequently measured by flow cytometry; %GFP cells are labeled in each histogram. **(B)** Quantification of NF-κB driven GFP reporter expression in transfected JPM50.6 cells as described in (A). Data are mean ± SEM for mean fluorescence intensity of GFP^+^ cells for 3 separate transfection experiments. Asterisks denote statistically significant differences (2-way ANOVA) between the stimulated groups indicated. **(C)** Immunoblots confirming the comparable expression of WT and mutant CARD1-FLAG proteins in cells from (A) at 24 h post-transfection. β-actin served as a loading control. Data are representative of 3 separate experiments. **(D)** WT Jurkat T cells were transfected with WT or mutant CARD11-FLAG plasmids as in (A). After 24 h, cells were stimulated with phorbol 12-myristate 13-acetate (PMA) for 20 min and lysed. Immunoprecipitations (IPs) using anti-BCL10 Ab were performed as previously described (12). BCL10 IPs and input lysates were separated by SDS-PAGE and immunoblotted Abs against CARD11, BCL10, and MALT1. **(E)** JPM50.6 cells were transfected with WT CARD11-GFP –/+ WT or mutant CARD11-FLAG plasmids as in (A). After 24 h, cells were stimulated with PMA for 20 min and lysed. FLAG IPs and input lysates were immunoblotted for total CARD11 protein; arrows indicate GFP- vs. FLAG-tagged CARD11. Actin served as a loading control for input lysates. IPs in (D,E) are representative of 2 separate experiments each. **(F)** PBMC from 2 healthy controls (HC) and patients III.1 and IV.1 were stimulated with PMA (20 ng/ml) plus monensin (2 μM) for 20 min. Cells were stained with FITC-conjugated mouse anti-human CD4 mAb, fixed in 1.5% paraformaldehyde and permeabilized in ice cold methanol before staining with AlexaFluor647-conjugated mouse anti-human phospho-p65 (Ser529) or AlexaFluor647-conjugated mouse anti-human IκBα. NF-κB activation was assessed in gated CD4^+^ T cells by flow cytometry; numbers in each histogram denote % of phospho-p65^+^ (left) or IκBα^hi^ cells (right). Data are representative of 2 separate experiments.

Affected patients displayed multiple clinical hallmarks of B cell Expansion with NF-κB and T cell Anergy (BENTA) disease, including (a) splenomegaly, (b) polyclonal B cell lymphocytosis, featuring elevated naïve/immature B cells with few class-switched memory B cells, (c) poor responses to pneumococcal vaccines, (d) bacterial/viral infections including pneumonias, molluscum contagiosium/warts, and moderate EBV viremia (2, 9). Because BENTA is caused by gain-of-function (GOF) variants in the lymphocyte scaffold molecule CARD11 (1, 4), we focused on *CARD11* as a potential candidate gene. Sanger sequencing was first performed on genomic DNA isolated from peripheral blood mononuclear cells (PBMC) from the index patient (IV.1) and his father (III.1). This analysis revealed a heterozygous four amino acid deletion and missense mutation within exon 5 of *CARD11* (NM_032415.3 c.701_713delinsT, p.His234_Lys238delinsLeu, hereafter referred to as H234LΔ235-8) (Figures 1B,C). The grandmother and great-grandmother were later confirmed to carry the same pathological mutation (Figure 1B). A CARD11 expression plasmid of this variant was constructed and expressed in a variant of the Jurkat human T cell line (JPM50.6) that lacks endogenous CARD11 expression and harbors an NF-κB-driven green fluorescent protein reporter (GFP) (13). Similar to a confirmed gain-of-function (GOF) CARD11 mutation (E134G) (4), H234LΔ235-8 induced constitutive NF-κB activation in unstimulated cells, increasing slightly upon TCR/CD28 crosslinking (Figures 2A,B). However, unlike E134G, co-expression of H234LΔ235-8 with WT CARD11 sharply reduced constitutive NF-κB activity. Strikingly, H234LΔ235-8 also significantly reduced WT CARD11-dependent NF-κB activation following TCR/CD28 stimulation (Figure 2B). Protein expression of WT, E134G and H234LΔ235-8 CARD11 were similar in transfected cells (Figure 2C). Co-immunoprecipitations in WT Jurkat transfectants revealed a substantial reduction of total CARD11 association with BCL10 and MALT1 following stimulation in the presence of H234LΔ235-8 but not WT or E134G CARD11 (Figure 2D). This decrease in CARD11-BCL10-MALT1 (CBM) complex formation was comparable, but slightly less pronounced, than that noted with a *bona fide* dominant negative (DN) CARD11 variant R47H (11). Moreover, FLAG-tagged CARD11 variants co-precipitated with WT GFP-tagged CARD11 when co-expressed in JPM50.6 cells, indicating WT CARD11 can directly associate with mutant CARD11 protein regardless of stimulation (Figure 2E). Taken together, these results suggest the H234LΔ235-8 CARD11 mutant can disrupt normal activation-induced CBM complex assembly and NF-κB signaling in T cells through DN interference. Similar to other patients harboring CARD11 DN variants, we also observed marked defects in NF-κB p65 phosphorylation and IκBα degradation in stimulated primary CD4^+^ T cells from patients III.1 and IV.1 relative to healthy control subjects (Figure 2F).

**Figure 3:**
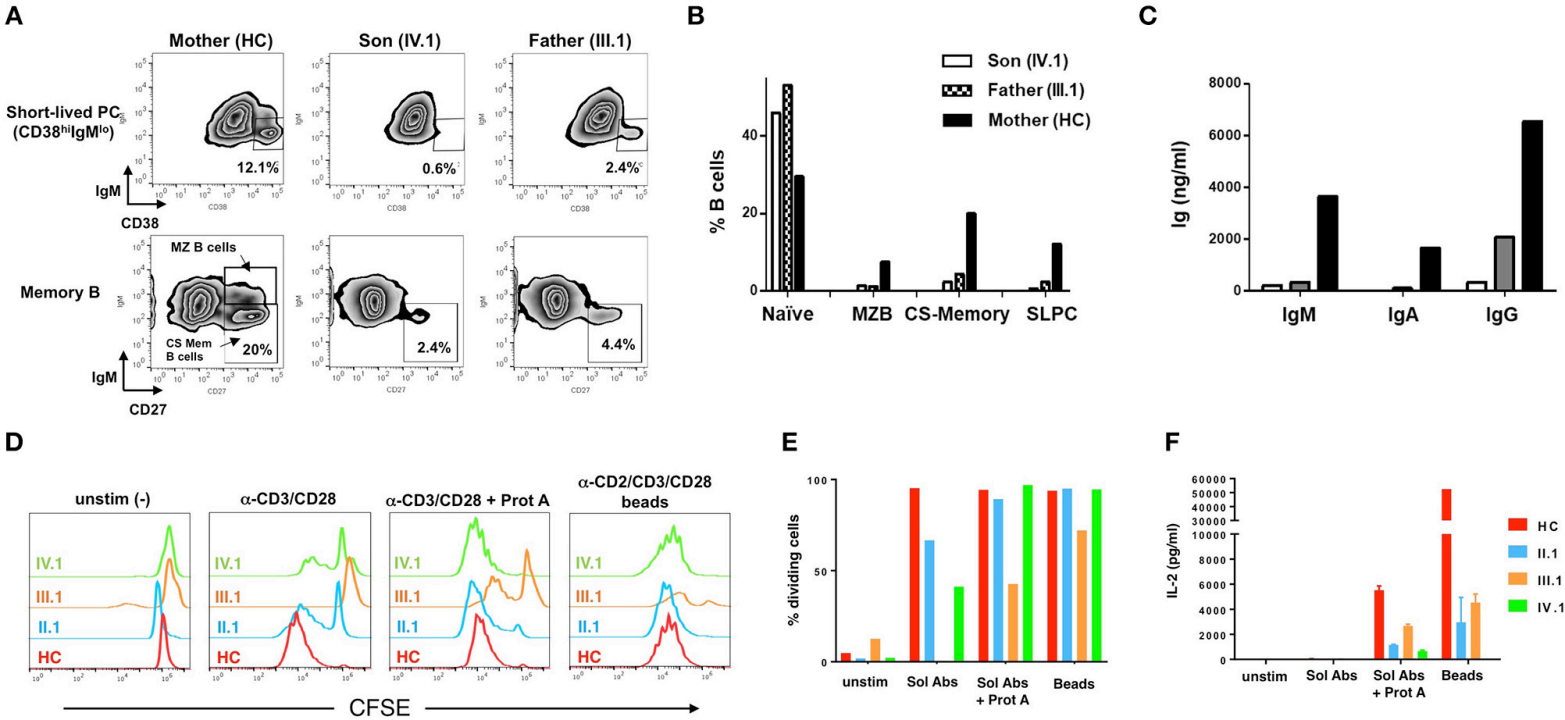
Impaired patient B cell differentiation and T cell hyporesponsiveness to polyclonal stimuli *in vitro*. **(A)** PBMCs from mother (healthy control), III.1 and IV.1 were cultured in cRPMI media with anti-CD40 (1 μg/mL) + IL-4 (200 U/mL) + IL-21 (50 ng/mL) at 37°C and 5% CO_2_ for 7 days as previously described (8, 15). B-cell subpopulations were analyzed by flow cytometry (Becton Dickinson LSRII, FlowJo Software) using Zombie aqua fixable viability dye and the following antibodies: PE-Cy7-conjugated mouse anti-human CD19, APC-conjugated mouse anti-human CD27, FITC-conjugated mouse anti-human CD38, PE-conjugated mouse anti-human IgM. Representative density plots are shown, with gates demarcating short-lived plasma cells (PC) (CD19+CD38+IgM–), class-switched (CS) memory B cells (CD19^+^CD27^+^IgM^−^) and marginal zone B-cells (CD19^+^CD27^+^IgM^+^). **(B)** Bar graph indicating the percentages of baseline naïve B cells (CD19^+^CD27^−^IgM^+^) and marginal, CS memory B and short-lived PC after *in vitro* differentiation in healthy mother and patients. **(C)** Immunoglobulin production in cell supernatants after 7 days *in vitro* stimulation of healthy mother and patients' PBMC with anti-CD40+IL-4+IL-21, quantified by ELISA. Data in **(A–C)** are representative of three independent experiments. **(D)** Healthy control, II.1, III.1, and IV.1 patient CD4+ T cells were labeled with 1 μM CFSE and stimulated with 2 μg/ml of soluble anti-CD3 and CD28 Abs ± Protein A (2.5 μg/ml), or MACS iBead particles (1:1 bead:cell ratio) loaded with biotinylated anti-CD2, anti-CD3, and anti-CD28 Abs (Miltenyi Biotec) in cRPMI for 5–6 days. Histogram overlays display T cell proliferation based on CFSE dilution on day 5. **(E)** Bar graph denoting the percentage of dividing T cells in each group in response to various stimuli. **(F)** IL-2 levels in day 6 T cell culture supernatants were measured by ELISA as previously described (4, 13). Data in **(D–F)** are representative of two independent experiments.

At baseline, the patient (IV.1) and his father (III.1) showed marked B-cell lymphocytosis: 51.6% and 44.5%, respectively, of their PBMC were CD19^+^ B-cells, compared to 13.8% for the patient's mother (healthy control, HC) and 10.2 ± 4.4% for a reference cohort. The majority were naive IgM^+^IgD^+^ B-cells (IV.1 74.9%, III.1 52.1%, HC 8.4%). To assess *in vitro* B-cell differentiation, PBMC (1 × 10^6^/mL) were cultured for 7 days with anti-CD40 (1 μg/mL), IL-4 (200 U/mL), and IL-21 (50 ng/mL) at 1 × 10^6^ PBMC/mL in complete medium [RPMI 1640 + 10% FBS, 2 mM glutamine, 1 mM sodium pyruvate, 15 mM HEPES buffer (pH 7.0), and 100 U/ml penicillin/streptomycin]. IL-21 promotes B-cell differentiation into class-switched (CS) memory and antibody-secreting plasma cells in the presence of CD40 signaling (14), and IL-4 enhances these effects (16, 15). Cells from patient (IV.1) and father (III.1) not only displayed poor short-lived plasma cell differentiation (Figure 3A, top panel) compared to the healthy mother (IV.1 0.6%, III.1 2.4%, HC 12.1%), but also decreased CD27^+^ memory B-cell differentiation including IgM^−^ CD27^+^ CS memory B-cells (IV.1 2.4%, III.1 4.4%, HC 20%) (Figure 3A, bottom panel). B cell differentiation phenotypes were quantified in Figure 3B. These results were associated with marked decreases in production of IgM (IV.1 0.2 mg/L, III.1 0.4 mg/L, HC 3.7 mg/L), IgA (IV.1 0.0 mg/L, III.1 0.1 mg/L, HC 1.7 mg/L), and IgG (IV.1 0.4 mg/L, III.1 2.1 mg/L, HC 6.5 mg/L) (Figure 3C). Purified T cells from affected family members showed impaired proliferation in response to anti-CD3/CD28 Ab stimulation *in vitro*, which was largely rescued by more robust stimuli (Ab crosslinking or Ab-conjugated beads) (Figures 3D,E). Nevertheless, patient T cells secreted less IL-2 in response to all stimuli (Figure 3F, Supplementary Figure 2). These results are consistent with previous work demonstrating that BENTA patient B-cells exhibit intrinsic differentiation defects despite enhanced survival *in vitro* (8), whereas T cells from both BENTA (CARD11 GOF) and CARD11 DN patients are hyporesponsive *in vitro* (4, 12).

## Discussion

Here we describe a unique four-generation family harboring a novel germline heterozygous indel mutation (c.701–713delinsT) in exon 5 of *CARD11*, leading to one altered amino acid and a deletion of 3 others (p.His234_Lys238delinsLeu). Clinical and laboratory findings suggested a relatively mild presentation of BENTA disease, based on a modest selective expansion of naïve/immature B cells that inversely correlated with age. Lackluster pneumococcal vaccine responses and moderate EBV viremia were also noted. Also consistent with BENTA disease, our *in vitro* assays also revealed profound defects in B cell differentiation and Ab secretion, as well as T cell hyporesponsiveness. Interestingly, members of this family also exhibited mild atopic manifestations (e.g., eczema, food allergy, elevated IgE) that waned over time. Collectively, we posit that these effects may result from dual GOF and DN effects of this unique mutation on TCR-induced NF-κB activation (Figure 2). However, in contrast to the family described here, T cells from patients with potent DN mutations generally proliferate poorly even after robust stimulation and strongly skew toward a Th2 phenotype, reflecting more severe defects in TCR-induced NF-κB and mTORC1 signaling (10–12). Although we suspect that the unique CARD11 variant described here is the major driver of shared phenotypes across four generations of affected family members, additional autosomal dominant gene variants may influence or modify disease presentation. Thus future whole exome sequencing (WES) analyses of this family could be informative.

Although CARD11 signaling is restrained by a complex set of redundant intramolecular interactions, single point mutations in the LATCH and CC domains can unlock an active signaling conformation without requiring AgR-dependent phosphorylation (17, 18). CARD11 must also oligomerize to signal (19), suggesting that both WT and mutant proteins should associate in shared CBM complexes (Figures 2D–E). For this unique variant, we speculate that the relative distribution of WT and mutant CARD11 molecules in these complexes may collectively allow for both constitutive NF-κB activation at steady state, *and* dominant interference of WT-dependent NF-κB signaling upon AgR engagement (Figure 2F). Nevertheless, the relative decline in disease severity observed with age (e.g., decreased B cell lymphocytosis, splenomegaly, and atopic symptoms) suggests the strongest phenotypic effects stemming from abnormal CARD11 signaling are ultimately overcome or somehow circumvented over time, as noted in both classic BENTA and CARD11 DN atopic patients.

The description of this family underscores some of the challenges in the clinical management of BENTA patients, including use of frequent boosters of conjugated vaccines, immunoglobulin replacement therapy, and increased likelihood of possible B cell malignancy (e.g., Hodgkin's lymphoma in II.1), particularly in the context of chronic EBV infection. Still, the family history described here suggests more aggressive clinical interventions to reduce the overall B cell burden (e.g., rituximab, immunosuppressants) are not warranted.

## Concluding Remarks

This first report of a family with blended BENTA and atopic symptoms further expands the spectrum of disease phenotypes that can be ascribed to a novel heterozygous *CARD11* variant. The signaling effects ascribed to this unusual mutation highlight the CC domain as a crucial governor of complex intra- and intermolecular CARD11 protein interactions. Despite the many congruent pathways linked to AgR ligation, aberrant CARD11 signaling appears to affect the proliferation, survival and differentiation of B and T cells differently. Clinically, the longitudinal perspective afforded here by four affected generations further suggests that although careful surveillance of such patients is essential, disease symptoms often improve over time.

## Ethics Statement

Patients' and biological parents' blood/tissue samples were obtained after informed consent through protocols established by the Lymphocyte Molecular Genetics Unit and approved by the Institutional Review Boards (IRB) of McGill University Health Centre and the National Institutes of Health (NIH). Experiments involving patient blood samples were performed at McGill University Health Center and Uniformed Services University conforming to IRB protocols.

## Author Contributions

MD and SA designed, executed and analyzed patient cell experiments, and wrote the manuscript. JRS, BD, and JS performed transfection experiments. BD performed biochemical and immunoprecipitation experiments. JRS and JN performed Sanger sequencing analysis. MR performed immunoglobulin heavy chain rearrangement analysis. AW, HM, and PA obtained consent and managed clinical care for patients at NIH. MD and BM provided clinical care at MUHC. HS provided clinical care at NIH. HS, BM, and AS supervised research studies and wrote and edited the manuscript.

### Conflict of Interest Statement

The authors declare that the research was conducted in the absence of any commercial or financial relationships that could be construed as a potential conflict of interest.
