# Supplementary material for: A Unique Heterozygous CARD11 Mutation Combines Pathogenic Features of Both Gain- and Loss-of-Function Patients in a Four-Generation Family
Source: Front Immunol. 2018 Dec 12;9:2944. doi: 10.3389/fimmu.2018.02944 (PMC6299974; doi:10.3389/fimmu.2018.02944)
Supplement: Supplementary file 1 [file Data_Sheet_1.pdf]

Supplementary Material: Desjardins et al.

Supplementary Figures & Legends

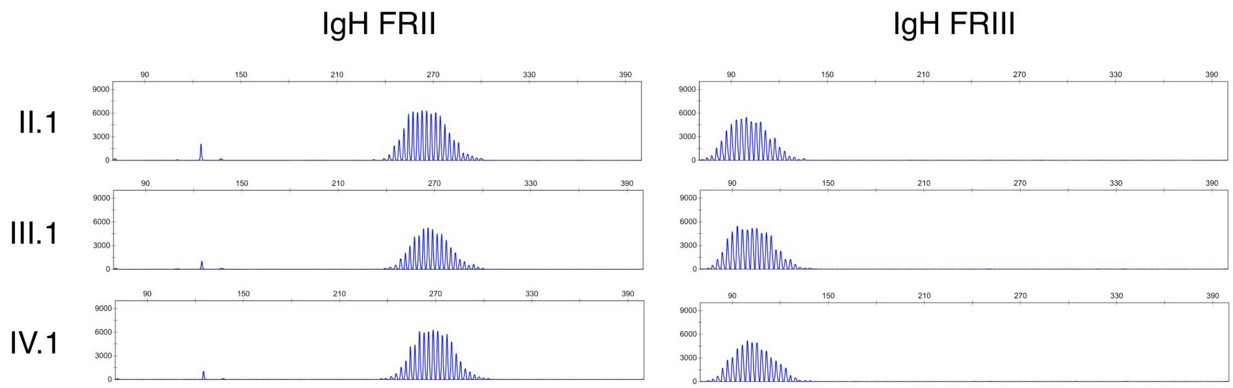

Supplementary Figure 1. IgH rearrangement indicates patient B cells are polyclonal. DNA was extracted from PBMC and PCR amplified for detection of immunoglobulin (IGH and IGk loci) and rearrangements as described in the Supplementary Methods. Polyclonal rearrangement patterns were detected in all reactions for patients II.1, III.1, and IV.1.

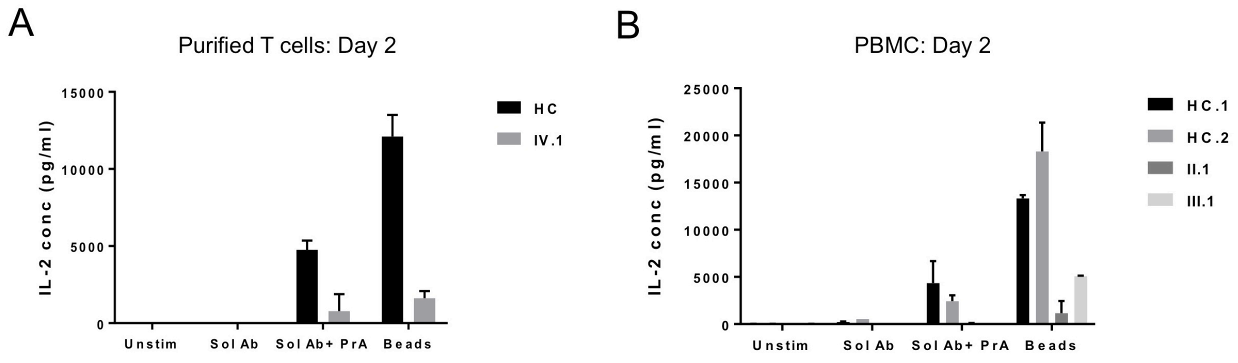

Supplementary Figure 2. Impaired IL-2 production by patient T cells. Purified CD4+ T cells (A) or PBMC (B) isolated from healthy controls or patients II.1, III.1, and IV.1 were stimulated with 2 µg/ml of soluble anti-CD3 and CD28 Abs ± Protein A (2.5 µg/ml), or MACS iBead particles (1:1 bead:cell ratio) loaded with biotinylated anti-CD2, anti-CD3 and anti-CD28 Abs (Miltenyi Biotec) in cRPMI for 2 days. IL-2 levels in day 6 T cell culture supernatants were measured by ELISA.

Supplementary Methods

Cells, tissues and treatments

Patients' and biological parents' blood/tissue samples were obtained after informed consent through protocols established by the Lymphocyte Molecular Genetics Unit and approved by the Institutional Review Boards (IRB) of McGill University Health Centre and the National Institutes of Health (NIH). Experiments involving patient blood samples were performed at McGill University Health Center and Uniformed Services University conforming to IRB protocols. Wild type (E6.1) and CARD11-deficient Jurkat T cells (JPM50.6) were maintained at 37°C, 5% CO_2 in RPMI 1640 medium (Lonza) supplemented with 10% fetal bovine serum (FBS, Sigma) and 100U/ml each of penicillin and streptomycin (ThermoFisher Scientific). Peripheral blood mononuclear cells (PBMC) were isolated from whole blood or buffy coats using Ficoll-Paque-Plus density gradient centrifugation as previously described. Subsequent to erythrocyte lysis using ACK lysis buffer (Lonza), $CD4^+$ T cells were isolated by negative selection using magnetic bead sorting kits (EasySep, StemCell Technologies).

B cell assays

PBMCs were cultured for 7 days at 1 x 10^6 cells/mL in complete medium [RPMI 1640 supplemented with 10% FBS, 2 mM glutamine, 1 mM sodium pyruvate, 15 mM HEPES buffer (pH 7.0) and 100 U/ml penicillin & streptomycin] with anti-CD40 Ab (1 μg/mL) ± IL-4 (200 U/mL) and IL-21 (50 ng/mL) at 37°C and 5% CO_2. B-cell subpopulations were analysed by flow cytometry (BD LSRII, FlowJo Software) after staining with: Zombie aqua fixable viability dye, PE-Cy7-conjugated mouse anti-human CD19, APC-conjugated mouse anti-human CD27, FITC-conjugated mouse anti-human CD38, PE-conjugated mouse anti-human IgM (BD Biosciences, BioLegend). Culture supernatant was collected and frozen at -80ºC for subsequent immunoglobulin (IgM, IgA and IgG) production assessment by ELISA (detection limit IgA: 0.977 ng/mL, IgG: 0.586 ng/mL and IgM: 15.6 ng/mL).

T cell assays

For proliferation assays, purified $CD4^+$ T cells were washed twice with PBS to remove any serum and resuspended in 500 µl of 1µM CFSE (CellTrace CFSE Proliferation Kit; Invitrogen) and incubated in 37ºC for 5-7 min. About 10 ml of complete media containing serum was added

to quench CFSE. The cells were then resuspended in complete RPMI, plated at a concentration of $1x10^6$ cells/well (200ul/well) in 96-well round bottom plates and stimulated with either 2 ug/ml each of anti-CD3 and anti-CD28 mAbs (BD Biosciences) ± Protein A (2.5 μg/ml), or MACS iBead particles (1:1 bead:cell ratio) loaded with biotinylated anti-CD2, anti-CD3 and anti-CD28 Abs (Miltenyi Biotec). IL-2 was measured in cell supernatants collected on days 2 or 6 post-stimulation by ELISA (ThermoFisher Scientific).

Intracellular flow cytometry

PBMC were stimulated with PMA (20 ng/ml) plus monensin (2 μM) for 20 min, then surface stained with 5 μg of FITC-conjugated anti-CD4 (Biolegend) for 30 min in ice. Cells were then fixed in 1.5% paraformaldehyde for 10 min and permeabilized in ice cold methanol for 20 min. - Cells were subsequently washed in FACS buffer (1X PBS, 1% FBS, 0.1% sodium azide) and stained for 30 min using AlexaFluor647-conjugated mouse anti-human phospho-p65 (Ser529) (BD Biosciences) or AlexaFluor647-conjugated mouse anti-human IκBα (Cell Signaling Technology). Gated $CD4^+$ T cells were analyzed by flow cytometry (BD Accuri C6, FlowJo software).

Cell transfections

An expression construct containing WT CARD11 driven by a hybrid EF-1/HTLV-1 promoter (pUNO-CARD11, Invivogen) was altered to include a C-terminal 3xFLAG tag as previously described (1). In some experiments, a CARD11-GFP fusion construct (pzCARD11-GFP) was also used (2). Site directed mutagenesis was performed to introduce mutations (H234LΔ235-8, E134G, R47H) using specific primers for linear amplification followed by DpnI digestion of methylated DNA. Mutations were verified by Sanger sequencing (USU BIC Genomic Facility). JPM50.6 cells ($5x10^6$/transfection) were transfected with 5-10 μg plasmid DNA using a BTX Electroporator (BTX Harvard Apparatus, 260 V/950 μF) and incubated in complete RPMI for 24 hrs at 37°C prior to stimulation with 1 μg/ml of anti-CD3 and anti-CD28 Abs. NF-κB-dependent GFP mean fluorescence intensity (MFI) was then analyzed at 24 hrs post-stimulation by flow cytometry (BD Accuri C6, FlowJo software). Statistically significant differences in GFP MFI were reported using two-way ANOVA tests (GraphPad Prism).

Immunoprecipitations and Western blotting

JPM50.6 and Jurkat cells transfected as described were lysed in 1% NP-40 lysis buffer as previously described (2). Lysates were separated on 4-20% Tris-Glycine gels (Bio-Rad), transferred to nitrocellulose (TransBlot Turbo, Bio-Rad), and blocked with 5% milk/TBS/0.1%Tween. Membranes were blotted using anti-CARD11 (1D12, Cell Signaling Technology), anti-BCL10 (A-6, Santa Cruz), anti-MALT1 (MT1/410, Abcam), anti-FLAG (M2) or anti-β-actin (AC15) Abs (Sigma). Bound antibodies were detected using appropriate HRP-conjugated secondary Abs (Southern Biotech) and ECL substrate (ThermoFisher) on a ChemiDoc imager (Bio-Rad). CBM complex immunoprecipitations (IPs) were performed as previously described using transfected cells ± 15 min PMA/ionomycin stimulation (3). Briefly, pre-cleared lysates were incubated overnight with 2 μg anti-BCL10 (A-6) or anti-FLAG (M2) plus 20 ml Protein G Sepharose beads (Sigma). Beads were washed 3x in lysis buffer and boiled in 2x Laemmli sample buffer (Bio-Rad) + 5% β-ME before gel electrophoresis and immunoblotting as described above.

Sanger sequencing of patient DNA

Genomic DNA was extracted from patient PBMC using a DNeasy Blood & Tissue Kit. The presence of the specific variant in exon 5 of *CARD11* (c.702_713delinsT) was detected by Sanger sequencing after PCR amplification using the following primers: CARD11 Ex5-F (5'-GGAAGCGTTGCCTTTTCTGAG-3'); CARD11-Ex5-R (5'-CTATCTCTGACAAATGGGT GGTG-3'). CLIA-certified Sanger sequencing was performed in the DNA Sequencing Laboratory of the Department of Laboratory Medicine, NIH Clinical Center. The clinical (diagnostic) sensitivity of Sanger sequencing performed using a capillary sequencer and current sequencing chemistries, as well as computer-assisted mutation detection, has been determined to be >99% for the detection of nucleotide base changes, small deletions, and insertions (4).

IgH rearrangement analysis

DNA was extracted from PBMC using a PSS USA Magnatron System 8LX automated DNA extraction robot and PCR amplified for detection of immunoglobulin (IGH and IGk loci) and rearrangements. Consensus primers were used to amplify framework regions II and III and the joining region of the immunoglobulin heavy chain gene (FRII-IGH, FRIII-IGH PCR)

as previously described (5), capable of detecting a clonal population of 3-10%. For both reactions the joining region primer was covalently linked to a fluorescent dye FAM to allow for fluorescence detection. The products were analyzed by capillary electrophoresis on an ABI 3130xl Genetic Analyzer, and electropherograms were analyzed using GeneMapper software version 4.0 (ABI).

Supplementary References

1. Brohl AS, Stinson JR, Su HC, Badgett T, Jennings CD, Sukumar G, et al. Germline CARD11 Mutation in a Patient with Severe Congenital B Cell Lymphocytosis. J Clin Immunol. 2015;35(1):32-46.
2. Snow AL, Xiao W, Stinson JR, Lu W, Chaigne-Delalande B, Zheng L, et al. Congenital B cell lymphocytosis explained by novel germline CARD11 mutations. J Exp Med. 2012;209(12):2247-61.
3. Ma CA, Stinson JR, Zhang Y, Abbott JK, Weinreich MA, Hauk PJ, et al. Germline hypomorphic CARD11 mutations in severe atopic disease. Nat Genet. 2017;49(8):1192-201.
4. Ellard S, Shields B, Tysoe C, Treacy R, Yau S, Mattocks C, et al. Semi-automated unidirectional sequence analysis for mutation detection in a clinical diagnostic setting. Genet Test Mol Biomarkers. 2009;13(3):381-6.
5. Ramasamy I, Brisco M, Morley A. Improved PCR method for detecting monoclonal immunoglobulin heavy chain rearrangement in B cell neoplasms. J Clin Pathol. 1992;45(9):770-5.
